# Heart-lung transplantation—global activity between 2003 and 2023, indications and outcomes

**DOI:** 10.1016/j.jhlto.2025.100405

**Published:** 2025-10-07

**Authors:** Jens Gottlieb, Charlotte Sybrecht, Are Martin Holm

**Affiliations:** a Respiratory Medicine, Hannover Medical School, Hannover, Germany; b German Center for Lung Research (DZL), Giessen, Germany; c Department of Respiratory Diseases, Oslo University Hospital, Oslo, Norway; d Institute for Clinical Medicine, University of Oslo, Oslo, Norway

**Keywords:** Heart-lung transplantation, Lung transplantation, Heart transplantation, Global health, Mortality

## Abstract

**Background:**

Historically, heart-lung transplantation (HLTx) was the primary treatment for end-stage cardiopulmonary disease. However, advancements in surgery and postoperative care have increasingly enabled the use of bilateral lung transplantation (LTx) instead. This article reviews recent global trends, indications, and outcomes of HLTx.

**Methods:**

Global transplant activity was analyzed using data from the European Committee on Organ Transplantation (CD-P-TO). Outcome data were drawn from international registries.

**Results:**

From 2003 to 2023, 103 countries reported transplant activity to CD-P-TO, covering a population that grew from 3.22 to 6.07 billion. Between 2019 and 2023, 27 countries reported performing HLTx, with 94 to 108 procedures annually worldwide—equivalent to 0.02 per million population (pmp). In comparison, isolated heart and lung transplants occurred at 1.42-1.75 pmp and 1.05-1.39 pmp, respectively.

In Europe, HLTx rates declined from 0.15-0.16 pmp (2003-2008) to 0.04-0.06 pmp (2019-2023). In the U.S., while in the US it slightly increased from 0.08-0.13 pmp to 0.13-0.16 pmp. Pulmonary vascular disease is now the leading indication for HLTx, with recipients averaging 15 years younger than those undergoing isolated thoracic transplants.

One-year survival after HLTx is still lower than LTx but slowly increasing. Long-term survival improves in those surviving the first year is better in comparison to isolated LTx.

**Conclusion:**

HLTx is currently performed in about one-quarter of reporting countries, accounting for just 1.3%-1.6% of thoracic transplant volume. Despite lower early survival, younger HLTx recipients who survive the initial year show promising long-term outcomes.

## Background

Combined heart-lung transplantation (HLTx) involves the simultaneous replacement of both the heart and lungs with those from a single deceased donor—a medical endeavor reserved for a highly select group of patients. Conditions such as congenital heart disease with secondary pulmonary complications, pulmonary arterial hypertension with severe right heart failure, and other rare dual-organ failure scenarios may warrant this combined transplant when no other therapeutic options remain viable.

Since the first successful heart-lung transplant was performed in 1981 at Stanford University, the surgical techniques, immunosuppressive regimens, patient selection criteria, and perioperative care have progressed significantly. HLTx remains among the rarest of solid organ transplants performed worldwide, in stark contrast to the thousands of isolated heart or lung transplants performed annually. The scarcity is driven by a complex interplay of clinical, logistical, and systemic factors, including limited donor availability, strict recipient eligibility criteria, and the need for specialized transplant centers with multidisciplinary expertise.

The landscape of HLTx continues to evolve in response to broader trends in medicine. Advances in mechanical circulatory and respiratory support technologies, such as extracorporeal membrane oxygenation and ventricular assist devices, have shifted the treatment paradigm for some patients who might previously have been considered for combined HLTx. Improvements in isolated organ transplantation and the management of comorbidities have further reduced the number of cases where dual-organ replacement is the optimal or only option.

Data on recent global thoracic transplantation activity is limited, aside from national reports and personal communications, to three main sources. The first is the International Society for Heart and Lung Transplantation (ISHLT), which publishes reports through its international thoracic organ transplant registry, covering heart and lung transplantation (LTx) activity from 2001 to 2019, with updated survival data released in 2023 (https://www.ishlt.org/registries/international-thoracic-organ-transplant-(ttx)-registry). The second source is the Global Observatory on Donation and Transplantation (GODT), available at www.transplant-observatory.org, which compiles worldwide data on organ donation and transplantation from 2008 to 2023 across 93 countries. However, this database does not specifically report on HLTx. The third source is the European Committee on Organ Transplantation (CD-P-TO), which coordinates the collection of international data on organ donation and transplantation in 103 countries, with annual reports including HLTx covering the years 2003 to 2023 available through its website (https://www.edqm.eu/en/organisation-work-programme-organs-tissues-cells).

This article explores the current global activity of HLTx in depth, regional variations in practice, statistical trends, and recipient outcomes. As we examine the state of HLTx today, we gain insight not only into a unique surgical frontier but also into the broader dynamics shaping global organ transplant policy and care delivery.

## Methods

A retrospective registry analysis was performed. The study was conducted following the ethical guidelines of the 1975 Declaration of Helsinki. Due to the anonymous nature of publicly available data and the absence of personal data collection, ethical approval was waived.

Data from the European Committee on Organ Transplantation (CD-P-TO) was selected as the primary data source for global activity analysis.^1^ Because it represents a large number of countries (*n* = 89 in the most recent report, representing 74% of the world population), covers the longest and most up-to-date time period, and has provided country-specific data since the beginning (Supplemental Table 1).

For this study, data were extracted from the Newsletter into a dedicated database, which included all participating countries along with their population data and thoracic transplant activity—specifically isolated heart, lung, and combined HLTx, including pediatric heart-lung procedures. Countries with continuous reporting 2003-2023 were displayed separately.

The Global Database on Donation and Transplantation publishes reports online in a collaboration between the World Health Organization and the Spanish Transplant Organization and was used for validation. For Australia, Canada, France, Germany, New Zealand, Spain, and the United States data from 2017 onward [for heart transplantation (HTx)] and from 2018 onward (for LTx) were compared between GODT and CD-P-TO.

A world map illustrating thoracic transplant activity was produced with MapChart (mapchart.net) and is used under the relevant creative commons licence. Countries were grouped into the following regions: North America, South America, Oceania, Arabia, Asia, Africa, and Europe. For more meaningful comparison, specific focus was placed on comparing North America, Oceania, Western Europe (Austria, Belgium, Denmark, Finland, France, Germany, Iceland, Ireland, Italy, Luxembourg, Netherlands, Norway, Portugal, Spain, Sweden, Switzerland, UK and excluding South-Eastern and Eastern Europe), and all other regions.

Outcome data and indications for HLTx were obtained from the ISHLT reports^2^, ^3^, ^4^ and the US-american results between 2013 and 2023.^5^ The most recent U.S. outcome data following isolated heart and LTx were extracted from the Scientific Registry of Transplant Recipients (SRTR).^6^, ^7^ In addition, outcome data after HLTx from Scandinavia (Denmark, Finland, Norway, and Sweden) were retrieved from Scandiatransplant upon request. SRTR-survival data is validated through quality assurance measures, whereas data from the ISHLT registry rely solely on self-reporting by transplant centers.

Statistical analysis was conducted using medians and interquartile ranges (25th and 75th percentiles) for metric variables, while categorical variables were presented as absolute numbers and percentages. Annual transplant activity was expressed as the number of procedures per million population (pmp).

## Results

Between 2003 and 2023, a total of 103 countries reported thoracic transplantation activity to the CD-P-TO, with participation varying from year to year. Among these, 35 countries—Austria, Belgium, Bulgaria, Croatia, Cyprus, Czechia, Denmark, Estonia, Finland, France, Germany, Greece, Hungary, Iceland, Ireland, Israel, Italy, Latvia, Luxembourg, the Netherlands, Norway, Poland, Portugal, Romania, Slovakia, Slovenia, Spain, Sweden, Switzerland, the United Kingdom, Australia, Canada, Georgia, and the United States—submitted data every year and thus provided a continuous record of thoracic transplant activity over the two decades. At the beginning of the reporting period in 2003, only these 35 countries contributed data, covering a population of approximately 3.22 billion. By 2023, the total population of reporting countries had increased to 6.071 billion. This expansion was largely driven by the start of continuous reporting from China in 2018 and from India in 2021. In contrast, Russia ceased reporting transplantation activity after 2020.

### Activity in HLTx and thoracic transplantation

**Figure 1:**
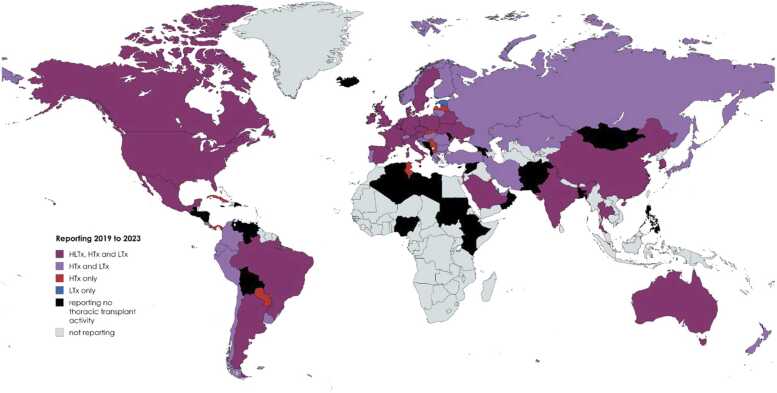
*World map of thoracic transplantation 2019-2023.* HTx, heart transplantation; LTx, lung transplantation; HLTx, combined heart-lung transplantation.

Between 2019 and 2023, a total of 96 countries reported transplant activity to the CD-P-TO. Of these, 66 countries (69%) reported at least one thoracic transplantation procedure (Figure 1). During this period, 43,602 heart transplants, 33,333 lung transplants, and 510 combined heart-lung transplants were reported. Specifically, 63 countries (66%) reported heart transplants, 55 countries (57%) reported lung transplants, and 27 countries (28%) reported heart-lung transplants. Moreover, 27 countries (28%) reported activity for all three types of thoracic transplantation (heart, lung, and combined heart-lung) consistently across the 5 years.

**Figure 2:**
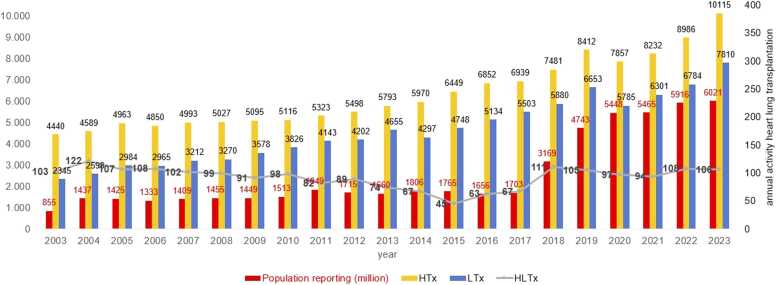
*Worldwide annual thoracic transplant activity and population of reporting countries.* HTx, heart transplantation, LTx, lung transplantation, HLTx, combined heart-lung transplantation.

Globally, between 94 and 108 HLTx were reported annually from 2019 to 2023, corresponding to an activity rate of 0.02 procedures per million world population (pmp) (Figure 2). Of these, 4 to 8 heart-lung transplants per year were performed in pediatric patients. For comparison, during the same time period, global HTx activity ranged from 1.42 to 1.75 pmp, while LTx activity ranged from 1.05 to 1.39 pmp.

**Figure 3:**
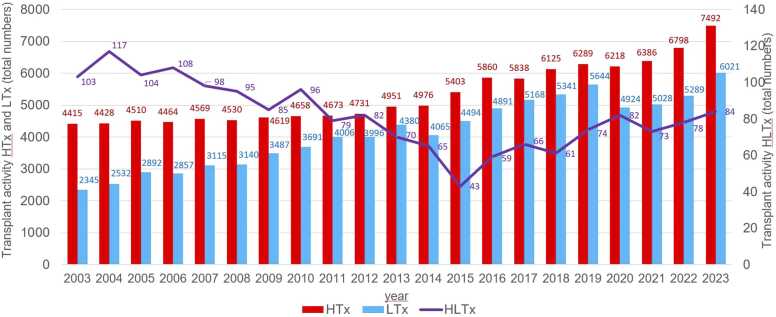
Annual heart-lung transplant and thoracic transplant activity in 35 countries* with continuous reporting 2003-2023. HTx, heart transplantation; LTx, lung transplantation; HLTx, combined heart-lung transplantation. * Austria, Belgium, Denmark, Finland, France, Germany, Iceland, Ireland, Italy, Luxembourg, Netherlands, Norway, Portugal, Spain, Sweden, Switzerland, UK and excluding South-Eastern and Eastern Europe.

**Figure 4:**
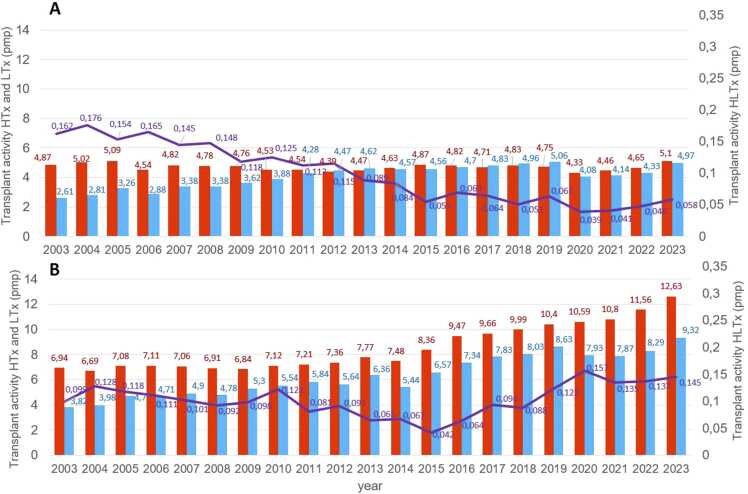
*Annual heart-lung transplant and thoracic transplant activity in North America (A) and Western Europe (B) 2003-2023.* HTx, heart transplantation; LTx, lung transplantation; HLTx, combined heart-lung transplantation; pmp, per million population.

In the 35 countries that reported consistently from 2003 to 2023, HLTx activity declined from 104 to 117 procedures per year between 2003 and 2007 to a low of 43 procedures in 2015 before gradually increasing again to 73-84 procedures annually again between 2019 and 2023 (Figure 3). Over this 20-year period, the combined population of these countries grew by 11%, from 854.3 to 948.9 million. Activity in HLTx within the past 20 years has declined in Europe since 2015 to a stable plateau of 0.039-0.069 pmp (Figure 4A), while in North America activity has slowly increased since 2015 from 0.042 to 0.145 pmp (Figure 4B).

Due to the low number of procedures, HLTx activity in Australia and New Zealand fluctuated significantly between 0.07 and 0.194 pmp. In other countries, activity was negligible, ranging from 0.004 to 0.008 pmp. In Western Europe, activity for isolated heart and LTx remained relatively stable from 2003 to 2023 (Figure 4A). In contrast, North America saw a marked increase in both procedures over the same period: HTx rose from 6.94 to 12.63 pmp, and LTx from 3.82 to 9.32 pmp (Figure 4B). In India, between 5 and 23 HLTx were reported annually between 2019 and 2023, while in China, the number ranged from 2 to 8 per year during the same period.

Forty-nine countries reported lung transplant activity (at least 1 procedure) to both CD-P-TO and GODT in 2023, with Sri Lanka reporting performing LTx only to GODT. For major countries represented in both sources—Australia, Canada, France, Germany, New Zealand, Spain, and the United States—data from 2017 onward (for HTx) and from 2018 onward (for LTx) were compared between GODT and CD-P-TO. No discrepancies were found, except in 2018, when GODT reported one additional lung transplant in Australia.

### Indications for HLTx

In the past, most heart-lung transplants were performed due to congenital defects with Eisenmenger syndrome (approx. 40%-50%), patients with various forms of pulmonary hypertension (approx. 25%-40%), and end-stage lung disease in combination with non-correctable cardiac conditions.^8^, ^9^ Improvements in medical therapy—particularly for pulmonary hypertension—and the less frequent transplant indication in cystic fibrosis have led to a slight shift in the spectrum of indications.

According to the 2019 ISHLT registry report, which included more than 2600 combined heart–lung transplants performed between 1999 and 2017, other forms of pulmonary hypertension were the most common indication worldwide (37%), followed by idiopathic pulmonary arterial hypertension (33%).^3^, ^4^ In the 2015 ISHLT registry report (covering 2004-2014, *n* = 763), congenital heart disease was listed as a separate entity for the last time; it accounted for 35% of indications, followed by idiopathic pulmonary arterial hypertension (28%), while other forms of pulmonary hypertension were not reported separately.^10^, ^11^ For comparison, the proportion of congenital heart disease was reported for the period 1982-2001 was also 35%. Subsequent reports adopted the diagnostic categories noted above, with congenital cases likely subsumed under non-idiopathic forms of pulmonary hypertension and possibly under “other indications.”

In the United States between 2013 and 2023, 36% of indications were classified as “other,” not further specified, while 10% were congenital (Table 1). Congenital heart defects were more frequently the indication for HLTx in the rest of the world (42%) compared with North America (33%) and Europe (36%) according to ISHLT.^3^, ^4^

**Table 1:** Patient Characteristics in the U.S. for Heart-Lung Transplantation in Comparison to Isolated Heart and Lung Transplantation

	UNOS 2013-2023, heart-lung *n* = 319^5^	SRTR 2018, 2023, *n* = 5571 lung recipients^6^	SRTR 2023, *n* = 4092^7^
Donor age, years	Mean 32 (standard deviation 12)	Median 33 (interquartiles 24-46)^#^	Median 30 (interquartiles 22-41)^##^
Recipient age, years	Mean 45 (standard deviation 12)	Median 59 (interquartiles 49-64)^#^	Median 59 (interquartiles 46-63)^##^
ECMO at transplant	26%	7%	8%
Diagnosis groups			
Obstructive (category A)	-	22%	-
Pulmonary vascular (category B)	46%	6%	-
Cystic fibrosis (category C)	3%	5%	-
Restrictive (category D)	5%	67%	-
Coronary artery disease	-	-	28%
Cardiomyopathy	-	-	62%
Valvular disease	-	-	1%
Congenital	10%	-	5%
other	37%	-	4%

Abbreviations: ISHLT, International Society for Heart and Lung Transplantation; SD, standard deviation; SRTR, Scientific Registry of Transplant Recipients; UNOS, United Network for Organ Sharing.

#SRTR 2015-2020,^17^ ##SRTR 1987-2018.^18^

Globally, between 1990 and 2002, 26% of all indications for HLTx were patients with cystic fibrosis, emphysema, or pulmonary fibrosis accompanied by heart disease, whereas between 2003 and 2017, this proportion declined to 17%.^3^, ^4^

There is limited data available on the characteristics of heart-lung transplant recipients compared to those receiving isolated heart or lung transplants.

Although donor age in the United States did not differ significantly from that in isolated heart or LTx, heart-lung transplant recipients between 2013 and 2023 had a mean age of 45 years—approximately 15 years younger than recipients of isolated transplants of either organ (Table 1). In data retrieved between 2000 and 2013 from the Scandiatransplant registry, the recipients of combined heart-lung transplants were significantly younger than those who received a double or a single lung transplant, with a median age of 35 years (interquartiles 25 and 45 years) compared to a median of 55 and 56 years, respectively (*p* < 0.0001).

Notably, the proportion of patients in the U.S. who required extracorporeal support at the time of HLTx was more than three times higher than for isolated heart or lung transplants, according to a current report.^5^

### Outcomes of HLTx compared to isolated heart and LTx

Survival outcomes for various sources for thoracic transplantation are shown in Table 2. According to the ISHLT Registry, one-year survival after HLTx has not significantly improved, remaining at 70% for the 2010-2017 cohort compared to 68% during 2002-2009. However, long-term survival showed an increase from 46% to 55% over the same periods. Conditional survival—defined as survival among patients who lived beyond the first year—was higher for heart-lung transplant recipients than for lung transplant recipients and was comparable to that observed after isolated HTx. The poor short-term outcome combined with the satisfactory long-term outcome is also found in the data from the Scandiatransplant registry.

**Table 2:** Recent Outcomes After Heart-Lung-Transplantation

				Survival rates		
Registry	Region	Period	Subjects, *n*	1 year	3 years	5 years
ISHLT heart-lung^3^, ^4^	Global	2010-2017	467	70%	59%	55%
ISHLT 2019 heart-lung^3^, ^4^	Global	2002-2009	743	68%	53%	46%
UNOS, heart-lung (Trefalls 2024)	USA	2013-2023	319	84%		60%
SRTR, lung^6^*	USA	2018-2022	13,056	89%	71%	60%
SRTR, heart^7^ *	USA	2016-2023	28,859	92%	86%	80%
Scandiatransplant^#^	Scandinavia	2000-2013	49	80%	62%	60%

Abbreviations: ISHLT, International Society for Heart and Lung Transplantation; SRTR, Scientific Registry of Transplant Recipients; UNOS, United Network for Organ Sharing.

*validated data, includes 1%-2% heart-lung recipients and 80% bilateral lung transplant recipients.

^#^Norway, Estonia, Denmark, Sweden, and Finland.

Interestingly, recent data from the United States report a 1-year survival rate of 84% and a 5-year survival rate of 60%, comparable to long-term outcomes following isolated LTx.^5^

Similarly, in the ISHLT registry, one-year survival rates increased from 65% to 70% across the periods 1992-2001 (*n* = 1619), 2002-2009 (*n* = 743), and 2010-2017 (*n* = 467), these differences were statistically significant between the period before 2002 and thereafter. The same trend was observed for 5-year survival, which significantly rose from 46% to 55% over the same periods.^3^, ^4^ Conditional survival among one-year survivors did not differ significantly across underlying diseases: five years after transplantation, 51%-53% of patients with pulmonary vascular diseases and cystic fibrosis were still alive, compared to 37% of those with restrictive lung disease and 42% with COPD.

In ISHLT reports early (typically within the first year) and late (typically after the first year) causes of death after HLTx were distinguished. In the most recent 2019 report, covering the period 1995-2018, the most frequent early causes of death within the first year were infections (19%), multiorgan failure (16%), graft failure (19%), and technical failure (15%).

According to the 2019 registry report, chronic graft failure (here defined as a combination of BOS or graft failure) accounted for about 35% of deaths late after HLTx and was the most frequent cause, followed by infections (∼25%), cardiovascular causes at 10%, and malignancies at about 6%. This spectrum does not differ significantly from long-term causes of death after isolated LTx.^3^, ^4^

According to the international registry, an encouraging proportion of 49% of heart-lung transplant recipients from the 1995 to 2004 cohort were reported by centers to be working after transplant; this proportion decreased to 27% in the 2005-2018 period.^3^, ^4^

## Discussion

While the total numbers of thoracic transplants has increased world-wide for both heart and lung transplants over the last decades, the number of combined heart-lung transplants has remained low at around 1 one percent of the number of heart transplants, although it seems to have recovered after a period of particularly low activity in the years 2013-2017.

Geographically, the distribution of combined heart-lung transplant activity is uneven, concentrated predominantly in North America, Western Europe, and select centers in Asia and Oceania. In some countries, only a handful of procedures have been done, while in others, such as the United States, the United Kingdom, Germany, and Australia, the numbers appear to be higher. It is not clear whether these differences reflect differences in healthcare infrastructure, organ donation systems, public health priorities, and resource allocation or rather reflect the existence of long-standing transplant programs with decades of accumulated experience Allocation for HLTx will be covered by a separate contribution in this JHLT Open series. In most Western countries, rare-donor thoracic organ offers like heart–lung blocks are prioritized and circulated to the highest-urgency candidates across domestic centers. In Europe, two supranational allocation systems—Eurotransplant and Scandiatransplant—coordinate cross-border exchange; Scandiatransplant policy explicitly includes heart-lung allocation, and Eurotransplant reports that 22.5% of all organs allocated in 2024 were exchanged across borders without specifying heart-lung blocks. Outside these networks, procurement organizations also collaborate to share deceased-donor organs internationally when no suitable domestic recipient is found. Such exchanges occur in North America (between US and Canada) and Europe. In the EU they are supported by Directive 2012/25/EU and are organized via a 24/7 platform for organ exchange called FOEDUS-EOEO. Results of FOEDUS-EOEO did not specify on the transborder exchange of heart and lung-offers.^12^ International sharing of heart and heart–lung offers remains constrained by the need for short ischemic times and donors after cardiac deaths in HLTx are used in less than 4% probably due technical challenges.^13^

The survival after combined heart-lung transplant seems to be inferior in comparison to isolated lung or HTx, although recent US reports were publishing encouraging results comparable to bilateral LTx. This difference was most pronounced during the first postoperative year, with a survival rate of 84% in the period from 2013 to 2023 compared with 66% in the ISHLT registry report from 1992 to 2017.^5^ The authors provided no explanation for this and improvement may be attributed to advances in surgical techniques and enhanced early perioperative management. Unfortunately, this U.S. publication does not distinguish between early and late causes of death. Moreover, it remains unclear whether validated survival data from SRTR were used, raising the possibility of reporting bias.

There are no comparative prospective studies across different indications between isolated transplantation and HLTx. Regarding outcomes between HLTx and LTx in patients with Eisenmenger syndrome, results are conflicting. In a retrospective analysis from Canada (34 patients after HLTx and 175 after isolated LTx), patients who underwent HLTx for Eisenmenger had better survival, whereas Scandinavian results (*n* = 57 after HLTx) showed no differences.^14^

In an analysis of 928 adult patients with idiopathic pulmonary hypertension who underwent transplantation, recorded in the SRTR registry between 1987 and 2012, 667 received bilateral lung transplants and 261 underwent HLTx. In this retrospective registry analysis, 1- and 5-year survival rates did not differ between the two procedures overall; however, survival was worse for bilateral LTx when patients had been hospitalized prior to transplantation.^15^

In two French centers, outcomes from 1996 to 2006 were retrospectively compared between patients who underwent HLTx (*n* = 79, 68% had pulmonary vascular diseases, mean age 36 years) and those who underwent isolated HTx (*n* = 141, 47% had cardiomyopathies and 28% had coronary artery disease, mean age 49 years).^16^ The 5-year survival rate after HLTx was 51%, compared to 69% after isolated HTx. The main causes of death after HLTx, similar to the ISHLT registry, were rejection (32%), infection (21%), and cardiac allograft vasculopathy (11%). Interestingly, the publication of U.S. data between 2013 and 2023 on the outcomes of HLTx reported that among 95 deaths out of 319 HLTx recipients, only 12% were due to infections, 3% to malignancies, and 18% to cardiovascular mortality. Chronic lung allograft dysfunction)was not listed separately as a cause of death.^5^ This highlights the objective traceability of mortality and causes of death in registries, such as the SRTR-Registry.^6^, ^7^

Since recipients of combined heart-lung transplants represent only a small fraction of thoracic transplants done, it is hard to accumulate enough data to perform meaningful comparisons of the outcomes of combined transplants and isolated double lung transplants, and for this study, we were not able to analyze in detail whether the combined transplant group were more urgently ill or whether there are other factors that may support the decision whether to list for a combined heart lung or an isolated lung transplant. In SRTR reports for isolated hearts or LTx also patients after HLTx were included 53 and 78 heart-lung recipients, but represented only 1% of the annual US-activity.

## Conclusion

Combined heart-lung transplants seem to have been largely replaced by bilateral lung transplants in most countries. Despite the observation that combined transplants may have a poorer short-term prognosis, in younger patients it may still be a justifiable option.

## CRediT authorship contribution statement

(I) Conception and design: JG, CS, MH. (II) Administrative support. (III) Provision of study materials or patients: all authors. (IV) Collection and assembly of data: JG, CS. (V) Data analysis and interpretation: JG, CS. (VI) Manuscript writing: all authros. (VII) Final approval of manuscript: all authors.

## Disclosure statement

JG and CS report no competing interests, AH serves as a chairman of Scandiatransplant.

## Footnotes

Appendix A Supplementary data associated with this article can be found in the online version at doi:10.1016/j.jhlto.2025.100405.
