# Supplementary material for: Heart-lung transplantation—global activity between 2003 and 2023, indications and outcomes
Source: JHLT Open. 2025 Oct 7;10:100405. doi: 10.1016/j.jhlto.2025.100405 (PMC12596949; doi:10.1016/j.jhlto.2025.100405)
Supplement: Supplementary file 1 — Supplementary material [file mmc1.docx]

Supplemental table S1: Sources of Data on International Heart–Lung Transplantation Activity, Indications and Outcomes

| Data source | European Committee on Organ  Transplantation of the Council of Europe (CD-P-TO), | Global Observatory in organ  transplantation | International Society for Heart and Lung Transplantation |
| --- | --- | --- | --- |
| Acronym | CD-P-TO | GODT | ISHLT |
| Website | <https://www.edqm.eu/en/organisation-work-programme-organs-tissues-cells> | [www.transplant-observatory.org](http://www.transplant-observatory.org) | <https://www.ishlt.org/registries/international-thoracic-organ-transplant-(ttx)-registry> |
| Reports published | online | online | peer reviewed journal <https://www.jhltonline.org/> |
| Period of annual reports | 2003-2024 | 2008-2024 | 2001-2019* |
| Heart-lung transplant activity reported | yes | not reported | yes |
| Lung transplant activity reported | yes | yes, in 2011 and from 2018 onwards | yes |
| Heart transplant activity reported | yes | yes, in 2011 and from 2017 onwards | yes |
| Number of Countries reporting^#^ | 91 | 93 | not reported (184 centers worldwide reporting heart-lung transplantation 1992-2017) |
| Indications and outcomes | not reported | not reported | yes |
| Country specific activity reporting | yes | yes, for thoracic transplants in 2011, and from 2017 onwards for heart transplants and from 2017 onwards for lung transplants | not reported |

*available slide sets, ^#^latest report 2024 (CD-P-TO) and 2023 (GODT)
